# Mapping Environmental Suitability of Scrub Typhus in Nepal Using MaxEnt and Random Forest Models

**DOI:** 10.3390/ijerph16234845

**Published:** 2019-12-02

**Authors:** Bipin Kumar Acharya, Wei Chen, Zengliang Ruan, Gobind Prasad Pant, Yin Yang, Lalan Prasad Shah, Chunxiang Cao, Zhiwei Xu, Meghnath Dhimal, Hualiang Lin

**Affiliations:** 1 Department of Epidemiology, School of Public Health, Sun Yat-Sen University, Guangzhou 510080, China; acharya@mail.sysu.edu.cn (B.K.A.); ruanzliang3@mail.sysu.edu.cn (Z.R.); yangyin3@mail.sysu.edu.cn (Y.Y.); linhualiang@mail.sysu.edu.cn (H.L.); 2 Institute of Surface-Earth System Science, Tianjin University, Tianjin 300072, China; 3 Department of Public Health, Manmohan Memorial Institute of Health Sciences, Kathmandu 44613, Nepal; gp.pant@gmail.com; 4 Center for Environmental and Occupational Health in Nepal (CEOHN), Karnali Academy of Health Sciences (KAHS), Mahalaxmisthan, Lalitpur 44511, Nepal; 5 Department of Health Services, Teku, Kathmandu 44600, Nepal; shahlalanprasad1971@gmail.com; 6 State Key Laboratory of Remote Sensing Science, Institute of Remote Sensing and Digital Earth, Chinese Academy of Sciences, Beijing 100094, China; caocx@radi.ac.cn; 7 School of Public Health, Faculty of Medicine, the University of Queensland, Herston, QLD 4006, Australia; xzw1011@gmail.com; 8 Nepal Health Research Council, Kathmandu 44600, Nepal; meghdhimal@gmail.com

**Keywords:** scrub typhus, suitability mapping, machine learning, Nepal

## Abstract

Being a globally emerging mite-borne zoonotic disease, scrub typhus is a serious public health concern in Nepal. Mapping environmental suitability and quantifying the human population under risk of the disease is important for prevention and control efforts. In this study, we model and map the environmental suitability of scrub typhus using the ecological niche approach, machine learning modeling techniques, and report locations of scrub typhus along with several climatic, topographic, Normalized Difference Vegetation Index (NDVI), and proximity explanatory variables and estimated population under the risk of disease at a national level. Both MaxEnt and RF technique results reveal robust predictive power with test The area under curve (AUC) and true skill statistics (TSS) of above 0.8 and 0.6, respectively. Spatial prediction reveals that environmentally suitable areas of scrub typhus are widely distributed across the country particularly in the low-land Tarai and less elevated river valleys. We found that areas close to agricultural land with gentle slopes have higher suitability of scrub typhus occurrence. Despite several speculations on the association between scrub typhus and proximity to earthquake epicenters, we did not find a significant role of proximity to earthquake epicenters in the distribution of scrub typhus in Nepal. About 43% of the population living in highly suitable areas for scrub typhus are at higher risk of infection, followed by 29% living in suitable areas of moderate-risk, and about 22% living in moderately suitable areas of lower risk. These findings could be useful in selecting priority areas for surveillance and control strategies effectively.

## 1. Introduction

Scrub typhus, an acute febrile zoonotic disease originating from Japan, is caused by bacteria called orientia tsutsugamushi 1899 [1]. It is transmitted through the bite of infected mites (the larval stage; known as chiggers) [2] and is prevalent in many countries in Asia-Pacific regions, extending from northern Japan and far-eastern Russia in the north, to northern Australia in the south, and Pakistan in the west [3,4]. Almost a million scrub typhus cases are reported annually, and a billion people living in this region are under the risk of the disease [4]. One recent report [5] claims that scrub typhus has been reported from well beyond the limits of the Tsutsugamushi Triangle and that has triggered concerns about the worldwide presence of scrub typhus [5,6]. In Nepal, scrub typhus has been a major public health problem with a series of outbreak across the country following the devastating earthquake of April 2015, with rising cases and spatial spread in the subsequent years [7]. 

Previous studies have reported the association of scrub typhus with climate, topography, vegetation dynamics, and socioeconomic factors. Suitable environmental conditions can provide ideal habitats for vectors to breed, become bacterium, survive long enough to become infectious, and finally transmit the disease to a susceptible human host [8]. For example, the spatial distribution of scrub typhus was associated with land use, NDVI, and relative humidity in southern India [6]. Positive association with temperature, relative humidity, and rainfall was also observed in southern China [9,10]. In Taiwan, the spatial pattern of the scrub typhus was positively associated with cropland, vegetation mosaic, and elevation [11]. Rainfall provides the moisture necessary for the survival and growth of host rodents and shows a positive association with rodent density [10]. The occurrence of scrub typhus is also linked to anthropogenic activities and socioeconomic factors. Farmworker population density and timber management are also positively associated with scrub typhus [12]. Changes in land use, animal populations, and climate, primarily due to increasing human populations, drive the emergence of zoonosis [13]. Elevated risk was also observed proximate to cultivated land [14]. A recent study in South Korea revealed a positive association between deforestation and scrub typhus [15]. 

Mapping the environmental suitability for disease transmission and identifying the associated environmental variables can provide crucial information for evidence-based decision making [16,17,18]. A better understanding of the spatial distribution of the incidence along with the possible associated factors of the distribution allows for more targeted disease control efforts and assist in the prediction of disease dynamics [16]. 

The ecological niche modeling (ENM) approach, machine learning algorithms, and GIS and remote sensing technology are playing an important role in disease mapping, identifying the associated environmental factors and advancing geography of infectious diseases [16,19]. There are several advantages of ENM predictive modeling in estimating disease distribution patterns over the traditional data-driven descriptive choropleth mapping and analytical spatial interpolation techniques [16]. ENM can produce a more accurate and robust map even with an incomplete and noisy dataset [16,20]. This is the most important advantage of the ENM in disease mapping as a collection of disease data is difficult due to underreporting, misdiagnosis, and ethical issues of personal identity and is costly and labor-intensive. Further, being non-parametric ENM are capable on describing nonlinear relationships between variables [9,21] to assess the interaction of spatial process of disease.

In the context of disease mapping, the aim is to determine habitat suitability for the persistence of a given disease agent and its transmission vectors at sufficient levels to result in human cases [16]. Generating disease maps from occurrence point data is thus similar to estimating species distributions, which characterize habitats suitable for a given species. Several algorithms are available to implement ENM, among which machine learning algorithms such as MaxEnt [22], and random forest (RF) [23] are superior due to their higher predictive accuracy.

Despite emerging evidence, the details of spatial distributions environmental suitability of scrub typhus transmission and associated environmental factors remain unclear in Nepal. Only a few studies have attempted to assess the environmental factors associated with occurrence and spread of scrub typhus [24,25,26]. However, no mapping efforts have been made yet to assess spatial variation of environmental suitable areas of scrub typhus transmission and associated factors in Nepal. In this study, we mapped the environmental suitability of scrub typhus and estimated human population under the risk of disease transmission in Nepal using the ecological niche approach and machine learning modeling techniques.

## 2. Materials and Methods 

**Figure 1:**
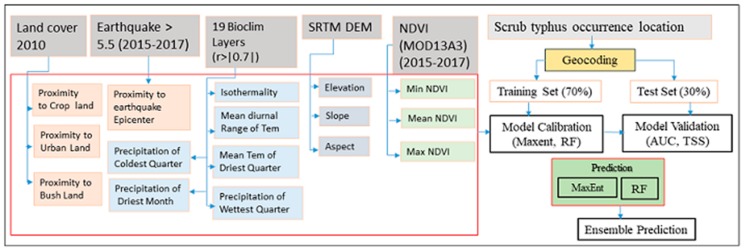
Flowchart summarizing the methodology used in this study.

The overall framework of this study included data collection, processing, the fitting machine learning model, model evaluation, and prediction and generation of the scrub typhus suitability map (Figure 1). We introduce the details of each step in the following sections.

### 2.1. Study Area

Nepal lies on southern slope of Himalaya between India and China in latitudes of 26°22′ N to 30°27′ N and longitudes 80°04′ E to 88°12′ E. It is a mountainous country with an area of 147181 square kilometers. Administratively, Nepal is composed of 7 provinces, 77 districts and 753 local bodies [27]. There is vivid land topography in Nepal with elevations ranging from 60 m in the southern plains to 8848 m at Mount Everest in the north. Based on the land topography, there are five distinct physiographic zones—high mountain, middle mountain, hill, Siwalik, and Tarai [28]. Climate of Nepal is broadly subtropical monsoon with distinct seasonality. Depending on the pattern of precipitation, Nepal has four distinct seasons: winter (December, January, and February), pre-monsoon (March–May), monsoon (June–September), and post-monsoon (October and November) [29]. Due to variations of altitude and topography, there are distinct microclimate zones from subtropical in the southern Tarai plain, temperate in the midland to the freezing nival climate in the north. Owing to different terrain and harsh climates, the northern area of Nepal is sparsely populated while the lowland southern Tarai and less elevated river valleys are highly populated.

### 2.2. Input Data 

#### 2.2.1. Scrub Typhus Occurrence Location 

**Figure 2:**
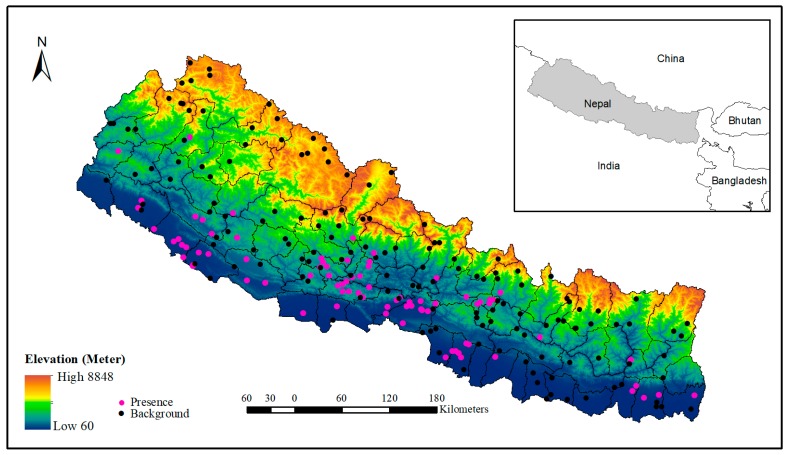
Location of the study area showing the elevation gradient and distribution of presence and background point of scrub typhus occurrence in Nepal.

We extracted the occurrence locations of scrub typhus from the line listing file received from the Epidemiology and Disease Control Division (EDCD), Government of Nepal. The line listing file contains the addresses of patients which include names of the VDCs/municipalities and their respective wards, including the district name. Then, the address data were geocoded using the “opencage” geocoder package (https://opencagedata.com/) in R. The “opencage” is an open access platform, which provides a geocoding facility up to 2500 addresses free of cost per day. In this process, we were able to geocode 123 villages/towns from where at least one case of scrub typhus had been reported in the government health system during 2015 to 2018. These scrub typhus occurrence locations (Figure 2) were spatially representative, covering almost entire country. 

#### 2.2.2. Environmental Covariates

We used a range of predictor variables in this study. These factors are environmental information relevant to disease amplification and transmission-related with scrub typhus including topographical, climatic, and vegetation dynamics [8,9,11,30]. Complete list of potential environmental variables used in the study is presented in Table 1. 

**Figure 3:**
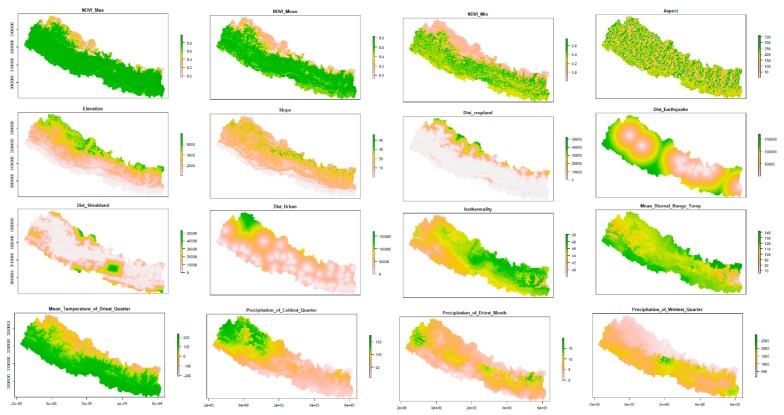
Spatial distribution of selected environmental factors used in mapping and modeling of scrub typhus disease risk in Nepal.

We used SRTM DEM with 90-m spatial resolution (http://srtm.csi.cgiar.org) to derive topographical variables including elevation, slope, and aspect. The DEM was directly used as a continuous elevation layer. For slope and aspect geo-processing, tools available in arc GIS were used to calculate topographic slope and aspect of the study area. We used the most widely used 19 bioclim layers from the WorldClim geoportal (http://worldclim.org) [31] to characterize climatic variations in the study area. We removed highly correlated variables with a threshold of Pearson correlation r >|0.7| and retaining five least correlated variables [32]. To characterize vegetation dynamics, we used monthly MODIS time series NDVI (MOD13A3) [33] from 2015–2018 synchronizing the time periods of scrub typhus geolocation data using the MODIStsp package [34]. We extracted three NDVI metrics NDVI_min,_ NDVI_mean,_ and NDVI_max._ In addition, we also extracted four proximity variables including distance from cropland, distanced from shrubland, distance from urban land and distance from earthquake epicenter with a magnitude higher than 5.4 for this study. The landcover map of 2010, which is publicly available in the geoportal of ICIMOD [35], was used to compute proximity of selected land covers while earthquake epicenters with magnitudes of 5.4 or higher that occurred in Nepal following the devastating Barpak earthquake till 2017 were retrieved from the NASA website (https://earthquake.usgs.gov). Euclidean distance function in Arc GIS 10.3 was used to compute the proximity for selected land cover and earthquake epicenter data. The spatial distributions of the selected environmental variables are presented in Figure 3.

### 2.3. Mapping and Modeling 

We applied MaxEnt and RF machine learning modeling techniques to identify different environmental factors and predict the spatial distribution of scrub typhus transmission risk in Nepal. These machine learning modeling techniques are robust due to their high predictive capacity [16]. Previous studies have widely used these methods in spatial modeling and prediction of different fields including spatial prediction and disease mapping [9,32,36,37]. 

MaxEnt estimates disease distributions by finding the distribution of maximum entropy: the simplest possible distribution that is consistent with the mean and variance of the observed distribution. RF is a tree-based classification and regression tree (CART) [38] algorithm. CART recursively partitions the environmental space into a large number of subsets within which separate regression models are fitted and then recombined to give a complex final response. The RF is the improved version of CART to address the overfitting problem through the bagging concept. RF builts trees using randomly selected bootstrap samples of the training data (used to build the model), with the number of bootstrap samples equal to the number of trees (ntrees) selected. Each tree is split by randomly sampling a number of predictor variables to use (mtry) at each node and then choosing the best split [23].

We used 89 geo-occurrence locations of scrub typhus and a set of environmental variables to fit the model. As running the modeling requires a background or absence location, we generated 153 background points using the randomPoint function of the “dismo” package covering the entire study area, keeping a proximity threshold of background points from the presence points (3 × 3 km) [39]. As an absence location which represents a location of the least likely occurrence of disease is normally difficult to collect [40], background points are usually used as alternatives in the modeling mapping of disease distribution. 

For an accuracy assessment, we divided the geo-occurrence points randomly into training and testing subsets in the proportion of 70 and 30 percent, respectively. The area under curve (AUC) of the receiving operating characteristics (ROC) and true skill statistics (TSS) metrics were used to evaluate the model accuracy [41]. The AUC measures the predictive performance of the model by comparing the model’s predictive ability to the random prediction, and values range from 0 to 1 where 0.5 indicates random prediction and higher values correspond to a better model [22]. The TSS compares the number of correct predictions, minus predictions attributable to random guessing. In other words, it is the sum of sensitivity and specificity minus 1. Its value ranges from −1 to +1, where +1 indicates perfect score, 0 indicates random performance, and values of 0.5 or higher are generally considered acceptable model performance [42]. To account for variation in the model results that can arise from an arbitrary data split, we fitted each model 10 times, using a different subset of geo_occurrence points and a different random assignment of training based on the cross-validation (CV) approach. 

The relative influence of different environmental variables during the model fitting process was assessed based on the AUC test score. Further, we created response curve plots for the most important variables to examine the nonlinear relationship between environmental variables and predicted scrub typhus transmission risk. Finally, mean spatial predictions of both modeling techniques based on the fitted model and selected 16 environmental variables were produced and exported in raster format. The spatial risk patterns of scrub typhus were visualized using ARC GIS based on the mean spatial prediction of these techniques. To assess the coherence of the selected modeling techniques in spatial prediction, Pearson’s correlation coefficient was calculated [37] using the 10,000 random points generated from the entire study area.

To estimate the human population exposed at different levels of risk, we overlaid the reclassified final suitability map derived from the ensemble technique using the natural breaks [43] in ARC GIS with human population raster data retrieved from WorldPop (http://www.worldpop.org.uk/) geoportal. 

## 3. Results

We fitted 100 models, 50 models in each technique based on 10-fold cross-validation. Both modeling techniques performed well with mean AUC value above 0.8 and mean TSS value above 0.6 in both training and test dataset simulations, indicating the robustness of the fitted models (Table 2). We observed insignificant variations in AUC and TSS between the training and test split. 

**Figure 4:**
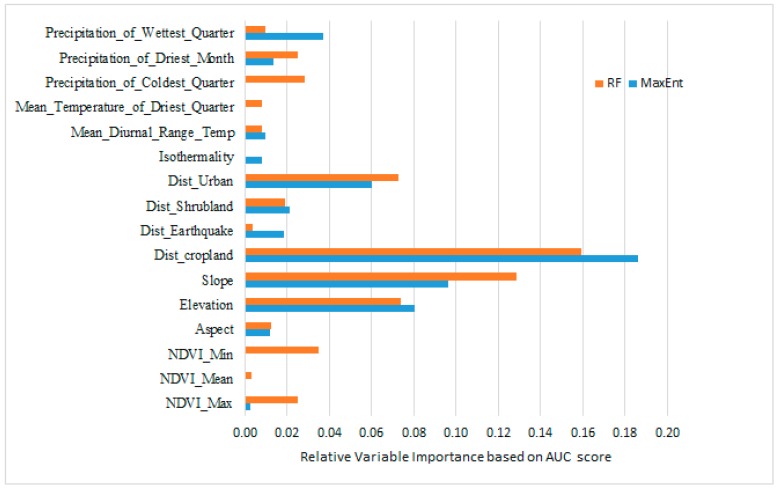
The relative importance of environmental variables in predicting the relative risk of scrub typhus.

Among the finally selected 16 geographic and environmental variables proximity to cropland, elevation, and slope and distance to urban land were the major contributors in both models, although rank of importance was little different depending on the modeling techniques (Figure 4). 

**Figure 5:**
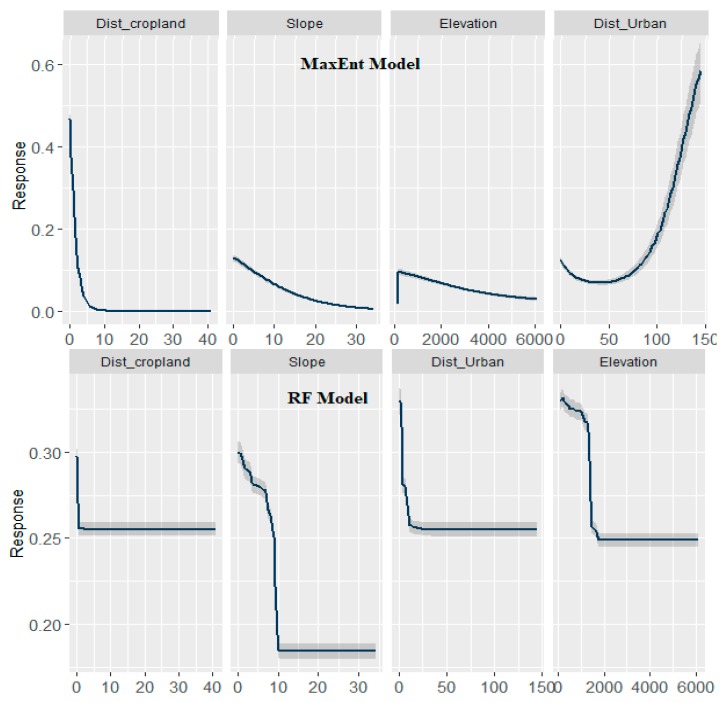
Marginal response curve plot of the four most important environmental factors for the occurrence of scrub typhus in Nepal. The *x*-axis shows the value of predictors, and the *y*-axis shows the value of predicted suitability. The shaded grey color shows the variability.

The negative association of proximity to cropland and proximity to urban land to the probability of occurrence of scrub typhus is observed for Nepal. The marginal response curve for proximity to cropland decreases sharply until the value reaches 800 m and no response is observed from then (Figure 5). However, the response curve of proximity to urban land is gentler and goes up to 1500 m. 

The marginal response of elevation is positive until the height reaches around 200 m and then it becomes negative. The negative association part of the curve is more smooth and has a response up to 6000 m asl. The risk of scrub typhus initially increases with an increase in rainfall but decreases gradually once rainfall reaches 43 mm. 

**Figure 6:**
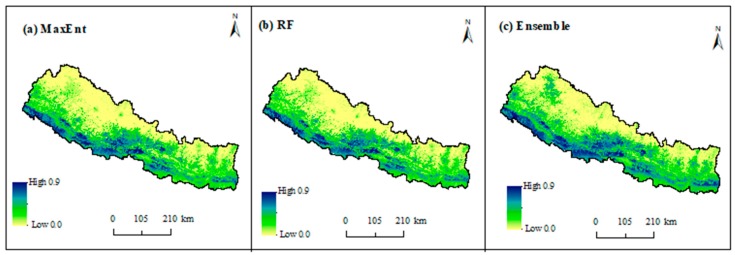
Probability of occurrence of scrub typhus in Nepal using the (**a**) MaxEnt, (**b**) random forest, and (**c**) ensemble methods.

Figure 6 depicts the spatial distribution of environmentally suitable areas of scrub typhus in Nepal based on MaxEnt, RF, and ensemble techniques where suitability values range from 0 (low) to 1 (high). In general, there is a broad consistency in both methods. Despite little variation in the prediction, both models are strongly correlated with Pearson correlation values higher (r = 0.8, *p* < 0.05) than when model predictions were validated based on 10,000 randomly generated sample points. The predicted high suitable areas are mainly distributed in lowland tarai and less elevated hill regions across the country in both models. The highly suitable areas are continuously distributed in the western tarai and the lower hills of central Nepal while it is irregular in the east, mainly in the southern region of east tarai. 

The results show that about 43% of the population of Nepal are currently living in the highly suitable areas of scrub typhus transmission in Nepal (Table 3) while 29% and 21% are living in suitable and moderately suitable areas. Only 6% of the population are living in the areas of environmental unsuitability for scrub typhus transmission. 

## 4. Discussion

Scrub typhus has been a major public health problem in Nepal since a 2015 outbreak across the country. About 3000 cases with 26 deaths were recorded in the last four years since its first outbreak [7,26]. The number of scrub typhus reported districts have also increased from 16 to 60 in the same period, showing a rapid geographic expansion of scrub typhus in Nepal [25]. In the context of rising incidences and expanding geographic distribution, scrub typhus is expected to replace typhoid as a common cause of febrile illness in Nepal [44]. This study assessed the spatial distribution of environmentally suitable areas of scrub typhus in Nepal using the ecological niche modeling approach, machine learning techniques along with reported cases of scrub typhus and a set of environmental and geographic explanatory variables. Further, this study also estimated the population living under different levels of risk. Our results revealed that both modeling techniques, in general, could be useful in identifying environmental factors and quantifying the areas susceptible to scrub typhus outbreak. However, ensemble prediction is more comprehensive and reduces prediction uncertainties compared to the single algorithms [45]. The spatial distribution patterns of environmentally suitable areas of scrub typhus disease were found to be largely influenced by the interaction of several environmental factors, including proximity to cropland, proximity to urban land, slope, and elevation. 

**Figure 7:**
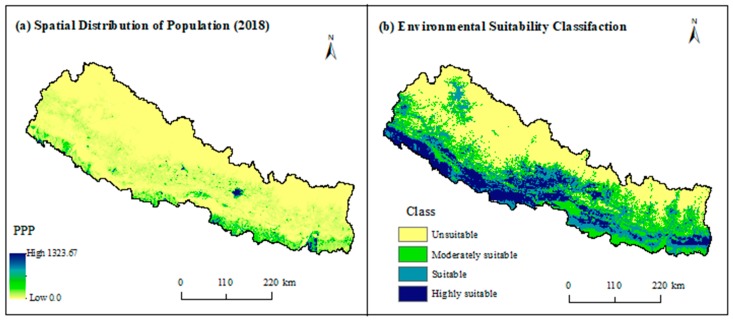
(**a**) Spatial distribution of population in Nepal in 2018 (people per pixel—PPP) and (**b**) environmental suitability classification of scrub typhus.

Our study shows that environmentally suitable areas of scrub typhus are widely distributed in Nepal mainly in the lowland Tarai and less elevated areas in mid-hills and mountains. These areas are major population concentration areas of Nepal (Figure 7). As a result, a significantly higher proportion of the population is under the risk of scrub typhus transmission despite a lower proportion of highly suitable areas compared to the total area of the country (Table 3). Previous studies also claimed these regions are high-risk areas of scrub typhus [7,46]. However, this is the first attempt that quantifies and maps its distribution in Nepal. Concurrent with these findings, about 81% of total reported cases were from lowland Tarai [7]. Similar to Nepal, subtropical southern districts of Bhutan have a higher risk of scrub typhus compared to the high mountain region in the north, although few cases have been recorded from throughout the country [47]. This indicates that lowland has a higher risk of scrub typhus; however, it might also occur in higher elevated mountains and hills. 

The spatial patterns of environmentally suitable areas are continuous in the west Tarai while it is irregular and patchy in the east. Unlike the west, our model predicts southern east Tarai as less suitable for scrub typhus. The possible reasons could be the lower elevation and gentler slope of this region. As a result, such areas remain wet and waterlogged for most part of the year, not favoring the growth and proliferation of the rodent population. The recent reports also showed a higher prevalence of scrub typhus in the west Tarai including Kailali and Kanchanpur districts and central hills, including Palpa, Syangja, and Tanahu districts [48]. 

Concurrent with previous studies, we found an elevated risk of scrub in the vicinity of cropland [14,25]. In line with our findings, the mosaics of cropland and vegetation are positively associated with the risk of scrub typhus in Taiwan [11]. The reason could be the availability of plenty of food essential for the proliferation of rodent hosts. A number of previous research have shown a positive association of scrub typhus with rodent density [10]. The reason could also be due to higher occupational exposure of farmers working in the cropland [49]. Previous studies also found that farmers work near the grassland scrubby vegetation and thus have higher chances of acquiring scrub typhus [25].

We found an inverse association between the probability of occurrence of scrub typhus and elevation in general; however, the association was positive up to around 200 m. Similar to our findings, the risk of scrub typhus incidence increases with an increase in elevation [11] in Taiwan. The possible reason of the inverse association might be the low temperatures in higher elevations. A decreasing trend of the proportion of cropland with an increasing altitudinal gradient in Nepal [50] might be another reason for the inverse association of elevation and probability of occurrence of scrub typhus. 

Slope is another important environmental factor of scrub typhus. Overall, there is a negative association between slope and the probability of scrub typhus occurrence. This association can again be explained by a decreasing trend of cropland with increasing slope gradients in Nepal. However, the association is much smoother in MaxEnt compared to the RF and BRT model. 

Although previous studies have suspected some association of scrub typhus with the proximity to earthquake epicenters [2,51] as the first worst outbreak occurred in Nepal immediately after the Gorkha earthquake of April 2015. The speculation was based on possible intimate contact between humans and rats that might have come out of their usual underground habitat after the earthquake. However, we did not find a significant role of proximity to earthquake epicenter with the occurrence of scrub typhus. 

Scrub typhus is generally a rural disease [2], and rural settings provide conducive environments for growth and proliferation of host and pathogen. However, in recent years, many urban cases have been reported from different countries including South Korea, Taiwan, and China [30,44,52]. In Nepal, urban cases of scrub typhus are also increasingly being reported [2]. The U-shaped marginal response curve of proximity to urban areas in the MaxEnt model indicates an elevated risk near the urban areas and far from the urban areas. All these indicate that the disease is no longer a rural disease and outbreak may occur in both rural and urban settings. Urban scrub typhus may have a significant impact because of the large population in urban areas.

Based on previous research findings [14], we included three NDVI layers of -minium, mean, and maximum- as potential predictors of scrub typhus distribution in Nepal. However, we did not find a strong predictive role of these variables in the occurrence of scrub typhus. Similarly, unlike previous studies, the role of climate factors was less important [14]. The possible reason could be due to the absence of proximity of variables in previous research or different ecological settings. 

This study has some strengths as well as limitations. This is the first spatially explicit scrub typhus research from Nepal, which has identified environmental risk factors and mapped the spatial distribution of disease transmission risk. The findings may help to close the knowledge gap on the spatial epidemiology of scrub typhus. The concerned health authority, including the Department of Epidemiology and Disease Control Division (EDCD), could use these findings to improve surveillance and control efforts targeting more locations that are predicted as the potential scrub typhus areas. However, due to the absence of complete disease data, we were unable to explore the data-driven exploratory analysis. Future studies are encouraged to focus on an exploratory analysis in one and the validation of filed data on the other. 

## 5. Conclusions

We assessed various environmental risk factors responsible for the occurrence and spread of scrub typhus, and mapped disease transmission risk for the first time in Nepal. Our results revealed that the environmentally suitable areas of scrub typhus in Nepal are widely distributed throughout the country with higher risks at lowland Tarai and the less elevated hills and mountains. Proximity to cropland and urban areas were the most important risk factors, followed by slope and elevation. Despite several speculations on scrub typhus and its association with proximity to the April 2015 earthquake epicenter, we did not find the earthquake’s important role in the distribution of scrub typhus risk in Nepal. 

## Figures and Tables

**Table 1:** Data source of environmental variables for the ecological niche model of scrub typhus.

Variable Category	Variables	Description	Sources
Topographical	Elevation	Elevation (m)	SRTM 90 m digital elevation data
	Slope	Slope(degree)	SRTM 90 m digital elevation data, http://www.csi.cgiar.org/, computed using the Slope Analysis tool in ESRI ArcGIS 10.2; a 1-km resolution dataset was generated
	Aspect	Aspect	SRTM 90 m digital elevation data, http://www.csi.cgiar.org/, computed using the Aspect Analysis tool in ESRI ArcGIS 10.2; a 1-km resolution dataset was generated
Climate	Bio2	Mean diurnal range of temperature	Worldclim Geoportal, http://worldclim.org/
	Bio3	Isothermality	Worldclim Geoportal, http://worldclim.org/
	Bio9	Mean temperature of driest quarter	Worldclim Geoportal, http://worldclim.org/
	Bio14	Precipitation of driest months	Worldclim Geoportal, http://worldclim.org/
	Bio16	Precipitation of wettest quarter	Worldclim Geoportal, http://worldclim.org/
	Bio19	Precipitation of coldest quarter	Worldclim Geoportal, http://worldclim.org/
Proximity	Dist2Urban	Distance to urban area (km)	Landcover map 2010, http://rds.icimod.org/Home/,computed using the Euclidean Distance Analysis and Zonal Statistics tool in ESRI ArcGIS 10.2 at 1-km resolution
	Dist2Cropland	Distance to cropland (km)	Landcover map 2010, http://rds.icimod.org/Home/,computed using the Euclidean Distance Analysis and Zonal Statistics tool in ESRI ArcGIS 10.2 at 1-km resolution
	Dist2Shrub	Distance to shrubland (km)	Land cover map 2010, http://rds.icimod.org/Home/,computed using the Euclidean Distance Analysis and Zonal Statistics tool in ESRI ArcGIS 10.2 at 1-km resolution
	Dist2Earthquake	Distance to earthquake epicenter (km)	Earthquake epicenter location between 2015–2017 with >5.5, https://earthquake.usgs.gov,computed using the Euclidean Distance Analysis and Zonal Statistics tool in ESRI ArcGIS 10.2 at 1-km resolution
NDVI	NDVI__min_	Minimum NDVI during the study period 2015–2018	MOD13A3, https://lpdaac.usgs.gov/dataset_discovery/modis/modis_products_table/mod13a3_v006, calculated minimum, mean, and maximum function in R
	NDVI__mean_	Mean NDVI during the study period 2015–2018	MOD13A3, https://lpdaac.usgs.gov/dataset_discovery/modis/modis_products_table/mod13a3_v006, calculated minimum, mean, and maximum function in R
	NDVI__max_	Maximum NDVI during the study period 2015–2018	MOD13A3, https://lpdaac.usgs.gov/dataset_discovery/modis/modis_products_table/mod13a3_v006, calculated minimum, mean, and maximum function in R

**Table 2:** Model performance comparison by area under the curve (AUC) of receiver operating characteristic curve (ROC) and true skill statistic (TSS) values.

Methods	AUC		TSS	
	Training	Test	Training	Test
**MaxEnt**	0.84	0.84	0.62	0.60
**RF**	0.86	0.86	0.65	0.58

**Table 3:** Estimated human population exposed at different levels of scrub typhus transmission suitability in Nepal.

Class of Suitability	Suitability Cut-of Values	Area (Km2)	Area (%)	Population	Population (%)
Unsuitable	<0.18	63,817.68	43.35	1,116,808	6.06
Moderately suitable	0.18–0.35	34,528.66	23.46	4,031,218	21.87
Suitable	0.3–0.5	27,758.33	18.86	5,395,633	29.27
Highly suitable	>0.52	21,076.31	14.32	7,887,215	42.79
Total		147,181	100.00	18,434,230	1000.00
